# Adaptively capturing the heterogeneity of expression for cancer biomarker identification

**DOI:** 10.1186/s12859-018-2437-2

**Published:** 2018-11-03

**Authors:** Xin-Ping Xie, Yu-Feng Xie, Yi-Tong Liu, Hong-Qiang Wang

**Affiliations:** 1 School of Mathematics and Physics, Anhui Jianzhu University, Hefei, 230022 Anhui China; 2 Institute of Intelligent Machines, Hefei Institutes of Physical Science, CAS, 350 Shushanhu Road, P.O.Box 1130, Hefei, 230031 Anhui China; 3 Present Address: School of Electronics and Information, Northwestern Polytechnical University, Xi’an, 710100 China

**Keywords:** Cancer biomarkers, Differential expression, Expression complexity, Regulation probability, Transcriptomics data

## Abstract

**Background:**

Identifying cancer biomarkers from transcriptomics data is of importance to cancer research. However, transcriptomics data are often complex and heterogeneous, which complicates the identification of cancer biomarkers in practice. Currently, the heterogeneity still remains a challenge for detecting subtle but consistent changes of gene expression in cancer cells.

**Results:**

In this paper, we propose to adaptively capture the heterogeneity of expression across samples in a gene regulation space instead of in a gene expression space. Specifically, we transform gene expression profiles into gene regulation profiles and mathematically formulate gene regulation probabilities (GRPs)-based statistics for characterizing differential expression of genes between tumor and normal tissues. Finally, an unbiased estimator (aGRP) of GRPs is devised that can interrogate and adaptively capture the heterogeneity of gene expression. We also derived an asymptotical significance analysis procedure for the new statistic. Since no parameter needs to be preset, aGRP is easy and friendly to use for researchers without computer programming background. We evaluated the proposed method on both simulated data and real-world data and compared with previous methods. Experimental results demonstrated the superior performance of the proposed method in exploring the heterogeneity of expression for capturing subtle but consistent alterations of gene expression in cancer.

**Conclusions:**

Expression heterogeneity largely influences the performance of cancer biomarker identification from transcriptomics data. Models are needed that efficiently deal with the expression heterogeneity. The proposed method can be a standalone tool due to its capacity of adaptively capturing the sample heterogeneity and the simplicity in use.

**Software availability:**

The source code of aGRP can be downloaded from https://github.com/hqwang126/aGRP.

**Electronic supplementary material:**

The online version of this article (10.1186/s12859-018-2437-2) contains supplementary material, which is available to authorized users.

## Background

Cancer is generally thought of to be driven by a series of genetic mutations of gene markers induced by selection pressures of carcinogenesis inside or outside cells [1, 2]. Such biomarkers, including onco- and tumor suppressor genes, often over-express or under-express in cancer cells as differentially expressed genes (DEGs), and are associated with uncontrollable proliferation or immorality of cancer cells [3]. With help of high throughput technology, one can screen out cancer biomarkers from transcriptomics data as DEGs between normal and cancer cells. However, transcriptomics data are typical of small sample, very noisy and inherently highly heterogeneous, rendering differential expression elusive. The heterogeneity of transcriptomics data remains a challenge for identifying cancer biomarkers [4, 5].

Over past decades, a large number of computational methods or tools have been developed for transcriptomics data analysis [6, 7]. Earliest is fold-change (FC) criterion, which, though simple and intuitive, ignores the heterogeneity and often outputs statistically and biologically unexplained results. Many sophisticated statistical tests have been developed for efficient identifications of DEGs, e.g. *t*-statistic and its various variants [8], Rankprod [9], cuffdiff [10], DESeq [11], DEGSeq [12] and edgeR [13]. Generally, these methods are categorized into two groups: parametric or non-parametric. The former often use a variant of *t*-statistic, e.g. SAM [14] and Limma [8], or negative binomial distribution, e.g., cuffdiff and DESeq, to model the differential expression of a gene. However, these methods made distribution assumptions that are often violated due to the complexity and heterogeneity of data in practice, and when applied to real data, they tend to produce similar overall results. Compared with the parametric methods, non-parametric methods generally do not make assumptions about data distribution but measure the difference of expression using a comparison-based quantity, e.g., ranks. The use of ranks relieves the harm from the expression heterogeneity to some extent. Among the non-parametric methods, commonly used is Rankprod proposed by Breitling et al. [9], which works well in many cases [15]. However, the performance of Rankprod depends on the proportion of differentially expressed genes and those in different directions, and it is computation-intensive due to the large numbers of sample comparisons involved, even computationally prohibited when sample size is very large. Recently, Nabavi et al. [16] introduced the Earth’s mover distance (EMD), a measure of distances commonly used in image processing, and developed a differential expression statistic named EMDomics. EMDomics relies on comparing the overall difference of the normalized distributions between two classes. EMDomics works well with data of moderate or larger sample size but can not tell about the direction or pattern of differential expression for a DEG. In summary, most of existing methods seldom consider or ignore the heterogeneity inherent in transcriptomic data and thus miss subtle but consistent expression changes [17, 18].

Although the difference in the average of expression between two sample classes are often employed in many transcriptomics analyses, such difference is not the only way that a gene can be expressed differentially [18]. Biologically, there exist a number of regulators or mediators in cells, e.g., transcriptional factors or miRNA, which, though work independently, regulate a target gene in a collective way and accordingly shape a complex and heterogeneous expression pattern across inter- or intra-classes for the target gene. Such regulatory mechanisms may account for the high biological variability where, for example, samples in one condition show a bimodal pattern of expression versus the other condition which show a unimodal pattern of expression across samples [16].

Relative to continuous gene expression space, gene regulation space is discrete and can simply consist of three discrete statues, i.e., up-regulated, down-regulated or non-regulated, and thus provides an alternative reduced representation for gene activity [19]. Generally, the heterogeneity of transcriptomics data comes from biological variability and non-specific technical noise, which can corrupt and contaminate differential expression signals of interest [20]. We here aim to address the problem of heterogeneity from a regulatory perspective by introducing regulation events, e.g.*,* up-regulation and down-regulation. The frequency of the regulation events occurring in samples not only reflects how genes are differentially expressed between two conditions but also contains information on how noise or contamination corrupts the data. Based on an unbiased estimator of the likelihoods of the regulation events, we developed a new differential expression statistic (aGRP), which can adaptively capture the heterogeneity of expression and makes it possible to flexibly detect cancer biomarkers with subtle but consistent changes. Because of no parameter pre-adjusted, the proposed method is also user-friendly and simple to use in practice. Experimental results on simulated data and real-world gene expression data demonstrated the superior performance of the proposed method in identifying cancer biomarkers over previous methods.

## Methods

For a given gene *g*, two regulation events can be defined between tumor and normal tissues: up-regulation, denoted by *U*, and down-regulation, denoted by *D*. If up-regulation *U* happens, it means that the gene has higher expression values in tumor than in normal tissues, while if down-regulation *D* happens, it means that the gene has lower expression values in tumor than in normal tissues. Let *P*(*U*) and *P*(*D*) represent the probabilities that events *U* and *D* occur between tumor and normal tissues, respectively. Considering the mutual exclusiveness between *U* and *D*, we formulate a regulation-based statistic, gene regulation probability (GRP), as the probability difference between the two events, namely

1 $$ T=P(U)-P(D) $$

The statistic *T*∈[− 1,1] reflects how likely the gene is differently regulated between the two conditions: The larger the absolute value of *T* the higher the likelihood of differential expression, and positive *T*s mean that an up-regulation event possibly occur in cancer while negative *T*s mean that a down-regulation event possibly occur in cancer. Biologically, genes with a positive *T* would be onco-gene-like while those with a negative *T* would be tumor suppressor-like. Note that *T* reflects an absolute quantity of regulation probability and can be completely rewritten as *T* = (*P*(*U*)-*P*(*N*))-(*P*(*D*)-*P*(*N*)) if considering the probability of non-significant regulation event (*P*(*N*)). We can estimate the two probabilities, *P*(*U*) and *P*(*D*), in a regulatory space in what follows.

### A simple estimator of *T* in a tri-state regulation space

For simplicity, consider a regulation space consisting of three statuses, i.e., up-regulated (1), down-regulated (− 1), and non-regulated (0). Assume *n* tumor samples and *m* normal samples. Let *a*_1*i*_ denote the expression level of gene *g* in the *i*th tumor, *i* = 1, 2, *…*, *n*, and *a*_2*j*_ the expression level in the *j*th normal sample, the expression profile of gene *g* can be denoted as *y* = [*a*_11_*, a*_12_*, …, a*_1*n*_, *a*_21_*, a*_22_*, …, a*_2*m*_]. We map the expression profile *y* into a tri-state regulation space as follows:

For the *i*th tumor sample with expression level *a*_1*i*_, the regulation status can be calculated as

2 $$ {r}_{1i}=\left\{\begin{array}{cc}1& {l}_i\ge \tau \\ {}-1& 1-{l}_i>\tau \\ {}0& others\end{array}\right. $$

where ${l}_i=\sum \limits_{k=1}^mI\left({a}_{1i}\ge {a}_{2k}\right)/m$ represents the proportion of normal samples that have an expression value not lower than *a*_1*i*_ in the total *m* normal samples, *I*(·) is an indicator whose value is 1 if the condition is true and 0 else, and the parameter *τ*, 0.5 ≤ *τ* ≤ 1, can be referred to as regulation confidence cutoff. Different values of *τ* can be preset to capture the varying heterogeneity of gene expression in practice.

Similarly, for the *i*th normal sample with expression level *a*_2*i*_, the regulation status can be calculated as.

3 $$ {r}_{2i}=\left\{\begin{array}{cc}1& {k}_i\ge \tau \\ {}-1& 1-{k}_i>\tau \\ {}0& others\end{array}\right. $$

where ${k}_i=\sum \limits_{k=1}^nI\left({a}_{2i}\le {a}_{1k}\right)/n$ represents the proportion of tumor samples that have an expression value not lower than *a*_2*i*_ in the total *n* tumor samples. As a result, a regulation profile of gene *g* across all the samples can be represented as

4 $$ R=\left[{r}_{11},{r}_{12},\dots, {r}_{1\mathrm{n}},{r}_{21},{r}_{22},\dots, {r}_{2\mathrm{m}}\right] $$

Based on the resulting regulation profile in Eq.(4), one can directly estimate the regulation probabilities, *P*(*U*) and *P*(*D*), using the total probability theorem. Take *P*(*U*) as example. Let *Y*_1_ and *Y*_2_ represent the sample spaces of tumor and normal classes respectively, we have

5 $$ P(U)=P\left({Y}_1\right)P\left(U|{Y}_1\right)+P\left({Y}_2\right)P\left(U|{Y}_2\right) $$

where *P*(*Y*_1_) and *P*(*Y*_2_) are the prior probabilities of tumor and normal classes respectively, and the two conditional probabilities, *P*(*U*|*Y*_1_) and *P*(*U*|*Y*_2_), can be estimated based on the regulation profile in Eq.(4) as

6 $$ {\displaystyle \begin{array}{l}\left\{\begin{array}{c}P\left(U|{Y}_1\right)=\frac{1}{n}\sum \limits_{i=1}^nI\left({r}_{1i}==1\right)\\ {}P\left(U|{Y}_2\right)=\frac{1}{m}\sum \limits_{i=1}^mI\left({r}_{2i}==1\right)\end{array}\right.\\ {}\end{array}} $$

Then, we have

7 $$ P(U)=\frac{s_u}{m+n} $$

where ${s}_u=\sum \limits_{i=1}^nI\left({r}_{1i}==1\right)+\sum \limits_{i=1}^mI\left({r}_{2i}==-1\right)$. Similarly, we have

8 $$ P(D)=\frac{s_d}{m+n} $$

where ${s}_d=\sum \limits_{i=1}^nI\left({r}_{1i}==-1\right)+\sum \limits_{i=1}^mI\left({r}_{2i}==1\right)$. As a result, a simple estimator of the regulation-based statistic *T* in the tri-state regulation space can be formulated as

9 $$ T=\frac{s_u-{s}_d}{n+m} $$

which can be referred to as GRP model. It can be noticed that the summation of *P*(*U*) and *P*(*D*), denoted by *S*, depends on the hard regulation confidence cutoff *τ*, i.e., *S* = 1 at *τ* = 0.5 but *S* < 1 at 0.5 < *τ* ≤ 1, and drops as τ increases.

### An unbiased estimator of *T* in regulation probability space

The simple GRP estimator in Eq.(9) uses a hard cutoff parameter to fit varying heterogeneities of gene expression in practice. However, no guidelines are immediately available for choosing the parameter in practice due to little or no knowledge on the heterogeneity of a given data set. To overcome the problem, we consider estimating *T* in a regulation probability space as follows. For calculating *P*(*U*), by removing the hard cutoff, we rewrite the conditional probabilities in Eq.(6) as

10 $$ \left\{\begin{array}{c}P\left(U|{Y}_1\right)=\frac{1}{n}\sum \limits_{i=1}^n{l}_i\\ {}P\left(U|{Y}_2\right)=\frac{1}{m}\sum \limits_{j=1}^m{k}_j\end{array}\right. $$

Compared with Eq.(6), Eq.(10) skips the empirical determination of regulation status in a sample and makes the conditional probabilities independent on an ad hoc hard cutoff. Essentially, this implies that regulation confidence cutoff is forcedly set to zero and that *P*(*N*) ≡ 0. As a result, an unbiased estimator of the occurring probability of the up-regulation event can be obtained, i.e.*,*

11 $$ P(U)=\frac{1}{n+m}\left(\sum \limits_{i=1}^n{l}_i+\sum \limits_{j=1}^m{k}_j\right) $$

and similarly, an unbiased estimator for the occurring probability of the down-regulation event is calculated as

12 $$ P(D)=\frac{1}{n+m}\left(\sum \limits_{i=1}^n\left(1-{l}_i\right)+\sum \limits_{j=1}^m\left(1-{k}_j\right)\right) $$

which is 1 minus *P*(*U*) as expected. Finally, according to Eq.(1), an unbiased estimator of *T* can be obtained:

13 $$ T=\frac{2}{n+m}\left(\sum \limits_{i=1}^n{l}_i+\sum \limits_{j=1}^m{k}_j\right)-1 $$

with *P*(*U*) + *P*(*D*) ≡ 1. The statistic in Eq.(13) can be referred to as an adaptive GRP model (*aGRP*), which explores more details on regulation information and can capture the intra-class or inter-class heterogeneity of expression in an adaptive way.

### Asymptotical significance analysis of aGRP

For simplicity, we consider the case of normal distribution data to provide an asymptotical significance analysis for the statistic *aGRP*. Supposing that the two groups of samples come from two normal distributions, i.e.*,*${Y}_1\sim N\left({\mu}_1,{\sigma}_1^2\right)$ and ${Y}_2\sim N\left({\mu}_2,{\sigma}_2^2\right)$, respectively, the follow probability distribution holds:

14 $$ P\left({Y}_1\ge {Y}_2\right)=\varphi \left(\frac{\mu_1-{\mu}_2}{\sqrt{\sigma_1^2+{\sigma}_2^2}}\right) $$

where $\varphi (x)={\int}_{-\infty}^x\frac{1}{\sqrt{2\pi }}{e}^{-\frac{t^2}{2}} dt$. Accordingly, the two regulation probabilities, *P*(*U*) and *P*(*D*), and the *aGRP* statistic all follow a normal distribution (see Additional file 1 for the detailed proof). Let $q=\varphi \left(\frac{\mu_1-{\mu}_2}{\sqrt{\sigma_1^2+{\sigma}_2^2}}\right)$, the unbiased estimator of *aGRP* in Eq.(13) follows a normal distribution, i.e.*,*

15 $$ N\left(2q-1,\frac{2\left({n}^2+{m}^2\right)q\left(1-q\right)}{nm{\left(n+m\right)}^2}\right) $$

Under the null hypothesis *H*_0_:*μ*_1_ = *μ*_2_, *aGRP* obeys the following normal distribution:

16 $$ N\left(0,\frac{\left({n}^2+{m}^2\right)}{2 nm{\left(n+m\right)}^2}\right) $$

which can be used to asymptotically estimate the significance for an observed *aGRP* in practice.

## Results

### Simple simulation data

We first evaluated the proposed method on simple simulation data. The simulation data contain two groups of genes:Group I consists of *G* = 1000 non-differentially expressed genes between two classes of samples while group II consists of *G* = 1000 differentially expressed genes. For group I, the expression values of genes in all samples were randomly sampled from standard normal distribution, while for group II, the expression values of genes in the two classes follow two normal distributions with different means (zero or 0.15) and the same deviation (0.1). Considering the influence of sample size, we varied the sample size of each class *n* = 6, 10, 20, 50, and in each scenario, twenty data sets were randomly generated and used for avoiding randomness on algorithm evaluation.

We compared the simple GRP and *aGRP* models on the simulation data. To investigate the property of *P*(*U*) and *P*(*D*), we plotted *P*(*U*) against *P*(*D*) for each gene on the simulation data. Results (Additional file 1: Figure S1) show that the GRP model had a complex joint distribution of *P*(*U*) and *P*(*D*): *P*(U) + *P*(D) =1 at *τ =* 0.5 but *P*(U) + *P*(D) < 1 at 1 ≥ *τ >* 0.5, and drops as *τ* increases, and in contrast *aGRP* favored a line *P*(U) + *P*(D) =1 as expected, suggesting the more favorable performance of *a*GRP. To examine the asymptotical significance analysis procedure of *aGRP*, we then compared the resulting *p*-values with those empirically estimated by permutation tests with randomly shuffled sample labels. Note that we considered *B* = 10, 50, 100, 1000 permutations of sample labels in the permutation tests respectively to gradually approximate the null distribution. It was revealed that the permutated *p*-values become closer to the asymptotic estimator as *B* increases (See Additional file 1: Figure S2), suggesting the justification of the derived significance analysis procedure.

**Fig. 1:**
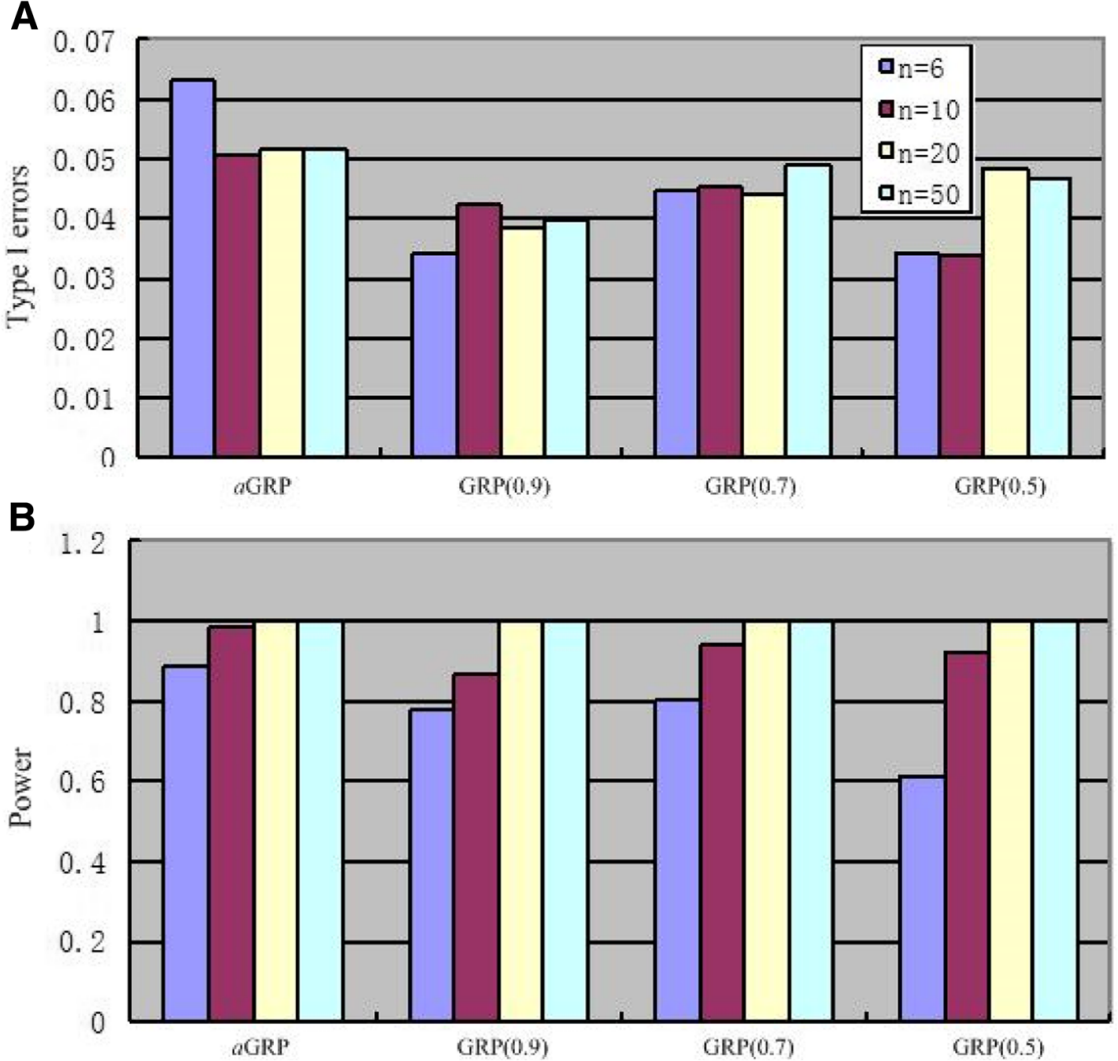
Average type I errors (**a**) and power (**b**) of *aGRP* and GRP models in different scenarios of sample size at an ad hoc *p*-value cutoff of 0.05 on Simple simulation data

We then investigated the type-I errors and power of the *aGRP* and GRP models based on the two groups of genes, respectively. Figure 1a barplots the average type-I errors at an ad hoc *p*-value cutoff of 0.05 by *aGRP* and GRP over 20 random data sets in each scenario of sample size. From a statistical perspective, the type-I error at an ad hoc *p*-value cutoff of 0.05 is expected to be 0.05. From this figure, it can be seen that *aGRP* had type-I errors closer to 0.05 than those by any of the GRP models in all the data scenarios. Figure 1b compared the powers of *aGRP* and GRP in identifying the *G* = 1000 differentially expressed genes at an ad hoc *p*-value cutoff of 0.05, showing that *aGRP* is more powerful than the GRP models, especially when sample size is small (*n* = 6 and 10).

### Simulated gene expression data

To evaluate the performance of *aGRP* on complex data, we next simulated gene expression data by revising the procedure in the reference [21]. The simulation data mimic real gene expression by forcedly adding hidden dependence structures, i.e.*,* correlation background. We assumed totally *G* = 10,000 simulation genes and divided them into 6000 non-differentially expressed genes between “tumor” and “normal tissue” and 4000 differentially expressed genes, of which one half up-regulated in tumor and the other half down-regulated. Let *n* be the sample size of each class, we generated a correlation background *X* [*G* × 2*n*] as follows: 1) randomly forming gene clumps of size *m*∈{1, 2, 3, ⋯, 100} and clump-wise correlation *ρ* from *U*(0*.*5*,* 1). 2) generating noise vectors *e.*_*j*_ of dimension *m* × 1 from *N*(0_*m*_,(1-*ρ*)*I*_*m*_ + *ρ*1_*m*_1’_*m*_) for sample *j*, *j* = 1*,*2, …,2*n*, and obtaining the background values of the *m* genes in the clump *x.*_*j=*_*μ + diag*(*ω*)*e.*_*j*_, where *μ* and *ω* are an *m* × 1 vector of elements μ_g_$\sim 1000{\chi}_5^2$ and of elements *ω*_*g*_ *= e*^*β*0/2^*μ*_*g*_^*β*1/2^ respectively. The correlation background increases the variability of data and makes the expression patterns heterogeneous. In the experiment, we set the parameters *β*_0_ = − 5, *β*_1_ = 2, and rendered the true expression ratios of DEGs to vary among $1+{2}^{-1/2}{e}^{\beta_0/2}{\delta}_g\sim U\left(1.29,1.58\right)$,*δ*_*g*_~*U*(1,2). To investigate the effect of sample size, we considered the four sample sizes *n* = 6, 10, 20 and 50, and as a result, four simulation data scenarios were obtained. In each scenario, 20 random data sets were generated and their average results were used for algorithm evaluation to overcome randomness.

We calculated the sensitivities, specificities, areas under the ROC curve (AUCs) and accuracies of *aGRP* at an ad hoc *p*-value cutoff of 0.05 in different scenarios of the simulated gene expression data. For comparison, we also applied previous methods, GRP models, Limma [8], SAM [14] and another popular non-parametric method, Rankprod [22], to analyze the simulation data. The previous methods, Limma, SAM and Rankprod, were implemented using the R packages Limma, siggenes, RankProd from Bioconductor, respectively. Note that for Limma, the proportional parameter was set as default. Table 1 lists the average performances of *aGRP* and the previous methods over 20 random data sets in each simulation scenario. From this table, we can clearly see that aGRP achieved higher accuracies than all the previous methods and comparable sensitivities and AUCs with Limma in almost all the simulation scenarios, showing the best overall performances of *aGRP*. Especially, aGRP is more advantageous for data scenarios of small (*n* = 6) or large (*n* = 50) sample size, and the higher sensitivities suggest the superior power of detecting subtle but consistent expression changes. For the GRP model, different settings of the regulation confidence cutoff led to similar results lying between those by *aGRP* and another non-parameter method, RankProd, as expected. Taken together, these results demonstrate the ability of *aGRP* in dealing with complex expression patterns for cancer biomarker identification.

**Table 1:** Performance (mean ± std.%) comparison among different methods on the simulated gene expression data

	Sensitivity	Specificity	AUC	ACC
n = 6				
Rankprod	33.24 ± 1.35	89.49 ± 0.91	70.11 ± 2.24	67.79 ± 0.94
Limma	39.73 ± 3.07	95.01 ± 1.99	78.54 ± 3.18	72.9 ± 2.59
SAM	32.95 ± 0.07	82.36 ± 6.68	70.02 ± 5.14	65.4 ± 4
GRP0.5	29.92 ± 2.13	**96.85 ± 1.0**7	78.48 ± 3.04	69.08 ± 1.61
GRP0.7	40.97 ± 0.05	94.06 ± 3.47	78.61 ± 2.67	71.73 ± 3.07
GRP0.9	42.99 ± 0.02	92.86 ± 1.03	77.98 ± 3.35	70.11 ± 3.62
aGRP	**43.45 ± 4.3**	93.16 ± 0.85	**80.08 ± 2.98**	**73.63 ± 2.51**
*n* = 10				
Rankprod	56.96 ± 1.34	85.48 ± 0.31	73.22 ± 0.85	73.27 ± 0.57
Limma	**57.04 ± 3.03**	95.49 ± 1.28	**88.32 ± 2.92**	**80.17 ± 1.77**
SAM	51.08 ± 3.05	77.9 ± 5.75	70.73 ± 4.56	68.73 ± 3.45
GRP0.5	47.05 ± 3.59	95.34 ± 1.65	85.42 ± 2.87	76.7 ± 0.99
GRP0.7	51.35 ± 3.58	95.16 ± 1.68	85.85 ± 2.98	77.89 ± 1.21
GRP0.9	51.01 ± 4.09	**96.35 ± 1.18**	85.87 ± 1.66	77.81 ± 1.71
aGRP	56.47 ± 3.4	96.16 ± 1.06	87.36 ± 2.67	79.7 ± 1.64
*n* = 20				
Rankprod	56.51 ± 1.29	85.4 ± 0.31	78.03 ± 0.92	73.84 ± 0.54
Limma	**86.84 ± 1.01**	95.30 ± 1.61	**96.02 ± 0.43**	91.06 ± 0.37
SAM	85.37 ± 0.1	92.45 ± 5.56	90.12 ± 3.73	86.46 ± 3.31
GRP0.5	80.5 ± 0.99	95.92 ± 0.92	94.00 ± 1.03	89.65 ± 0.87
GRP0.7	80.81 ± 1.58	**96.28 ± 0.73**	95.74 ± 0.85	89.97 ± 0.98
GRP0.9	80.69 ± 1.88	96.21 ± 1.02	94.43 ± 1.02	90.13 ± 0.85
aGRP	86.4 ± 1.7	95.70 ± 0.57	95.85 ± 0.5	**91.75 ± 0.94**
n = 50				
Rankprod	69.93 ± 0.69	80.07 ± 1.08	83.43 ± 0.92	76.08 ± 0.58
Limma	98.94 ± 3.9	95.95 ± 0.73	99.76 ± 1.01	96.57 ± 0.44
SAM	92.97 ± 0	89.36 ± 2.85	88.35 ± 1.51	90.82 ± 1.71
GRP0.5	97.16 ± 0.90	95.82 ± 1.01	99.51 ± 0.27	96.37 ± 0.25
GRP0.7	98.39 ± 0.47	95.43 ± 0.73	99.73 ± 0.16	96.56 ± 0.34
GRP0.9	97.06 ± 1.09	95.36 ± 1.04	99.54 ± 0.15	96.08 ± 0.92
aGRP	**98.96 ± 3.4**	**97.3 ± 0.85**	**99.85 ± 0.08**	**98.78 ± 0.51**

Best values are in bold

### Application to three real microarray data sets of lung cancer

Lung cancer is one of the most malignant tumors worldwide. We then applied the proposed method to identify gene signatures for lung adenocarcinoma (LUAD) based on three real-world lung cancer microarray datasets collected from GEO (http://www.ncbi.nlm.nih.gov/geo/): Selamat’s data (GSE32863), Landi’s data (GSE10072) and Su’s data (GSE7670). When generated, Selamat’s data used the HG-U133A Affymetrix chips for hybridization with 25,441 probes, Landi’s data the Illumina Human WG-6 v3.0 Expression BeadChips with 13,267 probes and Su’s data the Affymetrix Human Genome U133A array with 13,212 probes. All samples in the three datasets were divided into two classes, LUAD and normal tissue of lung (NTL). For the Selamat’s data, there are totally 117 samples, 58 of which are LUAD and 59 NTL samples; for the Landi’s data, there are totally 107 samples, 58 of which are LUAD and 49 are NTL samples; for the Su’s data, there are totally 54 paired LUAD/NTL samples. To preprocess the three datasets, we mapped probes into Entrez IDs and averaged the intensities of multiple probes matching a same Entrez ID to be the expression values of the gene, and adopted the coefficient of variation (CV) criterion with a CV cutoff of 0.05 to remove non-specific or noise genes.

**Fig. 2:**
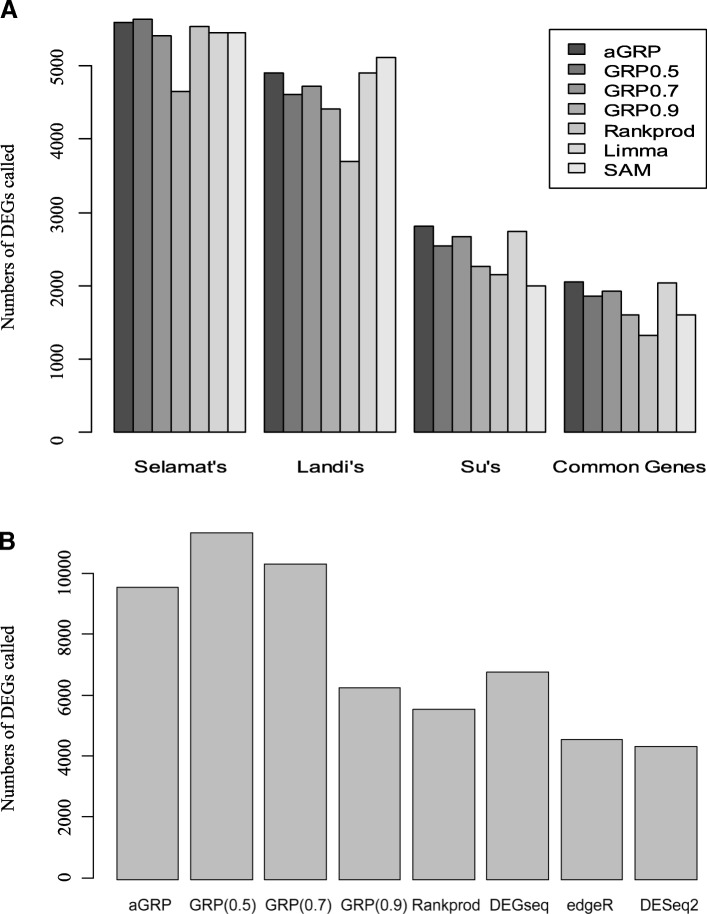
Comparison of the number of DEGs called among aGRP, GRP models and RankProd on the three LUAD data sets (**a**) and one HCC RNA-Seq data set (**b**). GRP0.5, GRP0.7 and GRP0.9 mean GRP models with τ = 05,0.7,0.9, respectively

We separately analyzed the three lung cancer data sets for identifying LUAD biomarkers in the experiment. To control false positive rates, the resulting *p*-values for each gene were corrected using the Benjamini-Hochberg (BH) procedure [21]. The previous methods, GRP, Rankprod [9], Limma [8] and SAM [14], were also applied to re-analyze these data sets for comparison. Figure 2a shows the numbers of DEGs called by these methods on each data set and the number of common DEGs across the three data sets at an ad hoc BH-adjusted *p*-value cutoff of 0.01. From this figure, we can clearly see that *aGRP* called more DEGs than those by the previous methods on almost all the three data sets and especially, most common DEGs across these data sets. This is consistent with the higher sensitivity on the simulation gene expression data (Table 1). For the GRP model, *τ* = 0.7 led to more DEGs than those of *τ* = 0.5 and 0.9 for two data sets, Landi’s and Su’s, while *τ* = 0.9 led to more DEGs than those of *τ* = 0.5 and 0.7 for Selamat’s data, implying the necessity of choosing proper *τ*s for different data applications for the GRP model. In contrast, *aGRP* adaptively captured the heterogeneity of data sets to automatically reach the optimal performance.

**Fig. 3:**
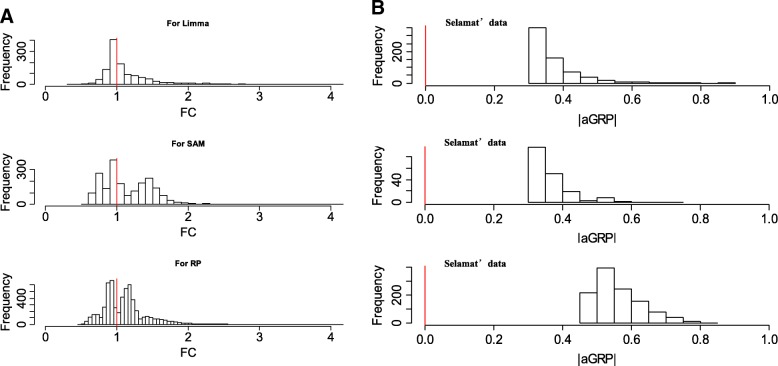
Distributions of FC (**a**) and aGRP statistics (**b**) of DEGs more called by aGRP than Limma, SAM or Rankprod on the three Lung cancer data sets

We further investigated the DEGs more called by aGRP than the previous methods, Limma, SAM and RankProd. Figure 3a shows the histograms of fold changes (FCs) of the DEGs for each of the three methods on the lung cancer data sets. For comparison, the aGRP statistics of these DEGs calculated on the three data sets were shown in Fig. 3b. It can be clearly seen that while the FCs are small with a distribution around one, the corresponding aGRP statistics are generally large, e.g., > 0.3, reflecting the high likelihoods of being regulated between tumor and normal tissues. We then looked into the biology of these DEGs by literature survey and found that many of them are associated with cancer. For example, gene PPP1R1A with a small FC of 0.97 but a large aGRP of 0.39 on the Selamat’s data is a tumor promoter, whose depletion can significantly suppress oncogenic transformation and cell migration. Differential expression of PPP1R1A was often observed in non-small cell lung cancers and colorectal cancers [23]. Luo et al.. [24] revealed that PPP1R1A-mediated tumorigenesis and metastasis relies on PKA phosphorylation-activating PPP1R1A at Thr35 in ewing’s sarcoma. Another gene CP110 with FC = 0.95 and aGRP = − 0.32 on Landi’s data was previously reported to be involved in lung cancers [25]. The inhibition of CP110 by MiR-129-3p are associated with docetaxel resistance of breast cancer cells [26] and centrosome number in metastatic prostate cancer cells [27]. Gene LRRC42 with FC = 1.45 and aGRP = 0.50 on Su’s data was extensively observed to be significantly up-regulated in the majority of lung cancers [28]. Taken together, these results demonstrate the special power of aGRP in capturing subtle but consistent changes of gene expression for cancer biomarker identification.

As described above, *aGRP* is featured with the ability of discerning DEGs regulated in different directions by the sign of the statistic *aGRP*. Totally, *aGRP* called 2023 common LUAD markers across the three data sets at an ad hoc BH-adjusted *p*-value cutoff of 0.01. We then divided the common DEGs into two categories: 1104 (Additional file 2: Table S1) with negative *aGRP* and 869 (Additional file 3: Table S2) with positive *aGRP*. According to the definition of *a*GRP, the former are likely down-regulated in LUAD relative to normal lung tissues as potential tumor suppressors. Take as an example TCF21 whose *aGRP*s are − 0.99, − 0.90 and − 0.99 on Landi’s, Selamat’s and Su’s data set respectively. Biologically, the gene encodes a transcription factor of the basic helix-loop-helix family, and has been previously reported to be a tumor suppressor in many human malignancies including lung cancer [29]. Recently, Wang et al.*...* [30] have reported that the under-representation of TCF21 is likely derived from its hyper-methylation in LUAD. The coordinated pattern of hyper-methylation and under-expression has been observed to be tumor-specific and very frequent in all types of NSCLCs, even in early-stage disease [31]. Smith et al. [29] used restriction landmark genomic scanning to check the DNA sequence of TCF21, consolidating the epigenetic inactivation in lung and head and neck cancers. Shivapurkar et al. [32] employed DNA sequencing technique to zoom in the sequence of TCF21, revealing a short CpG-rich segment (eight specific CpG sites in the CpG island within exon 1) that is predominantly methylated in lung cancer cell lines but unmethylated in normal epithelial cells of lung. We reason that the short CpG-rich segment narrowed down may be responsible for the abnormal down-regulation of TCF21 in LUAD.

On the other hand, the 869 markers with positive *aGRP* may be potential onco-genes for LUAD. Take as an example COL11A1 (*aGRP* = 0.92, 0.75 and 0.99 on Landi’s, Selamat’s and Su’s data set respectively). Biologically, the gene is a minor fibrillar collagen involved in proliferation and migration of cells and plays roles in the tumorigenesis of human malignancies. Recently, many studies observed that COL11A1 is frequently abnormally highly expressed both in NSCLC and in recurrent NSCLC tissues and suggested it to be a clinical biomarker for diagnosing NSCLC. Using NSCLC cell lines, Shen et al [33] witnessed the functional promotion of the gene COL11A1 in cell proliferation, migration and invasion of cancer cells, where the outcome of abnormal high expression of COL11A1 can be interceded by Smad signaling [33]. In addition, COL11A1 was also observed to over-express in ovarian and pancreatic cancer and to be an indicator of poor clinical outcome of cancer treatment [34]. Another markers worthy of noticing is HMGA1 with *aGRP* = 0.93, 0.80 and 0.98 on Landi’s, Selamat’s and Su’s data set respectively. Biologically, the protein encoded by the gene is chromatin-associated and plays roles in the regulation of gene transcription. HMGA1 was previously reported to frequently over-express in NSCLC tissues and to be associated with the metastatic progression of cancer cells. Using immunohistochemistry, Zhang et al [35] experimentally observed that high levels of HMGA1 protein are positively correlated with the status of clinical stage and differentiation degree in NSCLC, and suggested that HMGA1 may act as a convictive biomarker for the prognostic prediction of NSCLC.

To further assess the lung cancer markers identified by *aGRP*, pathway analysis was done based on functional annotation clustering analysis using DAVID, which is available at http://david.abcc.ncifcrf.gov/home.jsp. As a result, DAVID reported 38 KEGG pathways (Additional file 4: Table S3) that are significantly enriched in the list of total 2023 DEGs at an ad hoc *q*-value cutoff of 0.1. Literature survey showed that many of these KEGG pathways are related to cancer, e.g. cell cycle (Rank 1, *p*-value = 1.9 × 10^− 5^), extracellular matrix (ECM)-receptor interaction (Rank 2, *p*-value = 1.6 × 10^− 4^), and Pathways in cancer (Rank 11, *p*-value = 0.006). Of them, cell cycle comprises of a series of events that take place in a cell leading to the division and duplication of DNA. The pathway, Complement and coagulation cascades (*p*-value = 5.1 × 10^− 4^), has been recently reported to dysfunction in lung cancer [36]. The analysis also reported another two lung cancer-related pathways, PI3K-Akt signaling pathway (*p*-value = 0.009) and small cell lung cancer (*p*-value = 0.017). Biologically, the former regulates many fundamental cellular functions including proliferation and growth. There exist many types of cellular stimuli or toxic insults which can activate the signaling pathway. When activated, the pathway first employs PI3K to catalyze the production of PIP3 and then PIP3 as a second messenger to activate Akt. An active Akt can phosphorylate substrates that are involved in many vital cellular processes such as apoptosis, cell cycle, and metabolism, which play important roles in tumorigenesis of cells. Accumulated evidences indicate that the PI3K-AKT signaling pathway plays an essential role in lung cancer development. For example, Tang et al. [37] experimentally observed that Phosphorylated Akt overexpression and loss of PTEN expression in non-small cell lung cancer and concluded that the activity of the pathway confers poor prognosis. Recently, many clinical strategies have been suggested to target PI3K-AKT signaling pathway for clinical treatment of lung cancer [38], including the novel anticancer reagent sulforaphene [39]. In addition, Wang et al. [40] reported the role of PI3K/AKT signaling pathway in the regulation of non-small cell lung cancer radiosensitivity after hypo-fractionated radiation therapy.

### Comparison of consistency between aGRP and GRP

**Fig. 4:**
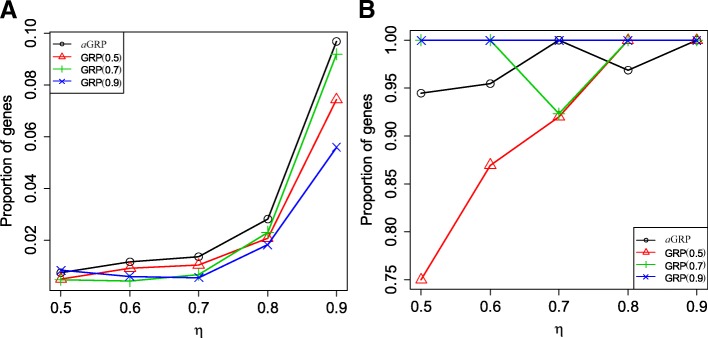
Changes of proportions of intersection genes (**a**) and genes with the same regulation direction (**b**) by aGRP and GRP across the three LUAD data sets with η. GRP 0.5, GRP 0.7 and GRP 0.9 are for the GRP model with τ = 05,0.7,0.9, respectively

Both *aGRP* and GRP are a regulation-based statistic for cancer biomarker identification, whose absolute values and signs indicate the strength and direction of regulation respectively. In the LUAD application, each marker were identified with three values of *aGRP* (or GRP) derived from the three data sets. Consider the same LUAD topic of the three data sets, the consistency or similarity among the results can be used to evaluate the reasonability and reproducibility of these regulation-based statistics. For this purpose, we divided the range [0.5,1] into five intervals, [η, η + 0.1], η = 0.5,0.6,0.7,0.8,0.9, and determined the genes whose absolute *aGRP*/GRP fall within each interval. Figure 4a compares the proportions of common genes in the union across the three data sets in each interval between *aGRP* and GRPs with *τ* = 0.5, 0.7, 0.9. From this figure, we can clearly see that both *aGRP* and GRP had a tendency of the proportion of common genes gradually increasing with η, showing the reasonability of regulation-based statistics. Compared with GRP, *aGRP* led to the higher proportions, irrespective of interval used, suggesting the better consistency of results by *aGRP*. We further compared the proportions of genes with a same regulation direction in the common genes across the three data sets between *aGRP* and GRPs in each interval, as shown in Fig. 4b. From Fig. 4b, it can be clearly seen that *aGRP* achieved the proportions larger than 94.44% (at η = 0.01) on all the intervals, confirming the consistency of the results by *aGRP*. Although the GRP model with *τ*= 0.9 had all the proportions of one, the proportions of common genes obtained by it were far lower than those by *aGRP* in all the intervals (Fig. 4a). Taken together, these results demonstrated the robustness and reliability of *aGRP* in cancer biomarker identification. The advantage of *aGRP* should be related to the ability of adaptively capturing the heterogeneity of expression across data sets.

### Application to RNA-seq expression data

We also evaluated the proposed method on RNA-seq expression data. Hepatocellular carcinoma (HCC) is the third leading cause of cancer-related deaths. We downloaded a HCC RNA-seq data set from the GEO database: Yang’s data (GSE77509) [41], which were measured using Illumina Hiseq 2000. All the samples in the data set consist of 17,501-gene expression profiles of 40 matched HCC patients and adjacent normal tissues. For quality control, we preprocessed the dataset by averaging the raw counts with a same Entrez ID as the expression levels of the corresponding gene. For comparison, we also applied three previous count-based method, DEGSeq [12], DESeq2 [42] and edgeR [13], besides the GRP model and Rankprod as above, to analyze the RNA-seq data in the experiment.

We first examined the similarity between the statistics of *a*GRP and DEGSeq on the RNA-seq data. As a result, the Spearman correlation of the *a*GRP statistic and log2 fold change from DEGSeq and the Spearman correlation of *p*-values derived from *a*GRP and *p*-values derived using DESeq are 0.86 and 0.617, respectively. Both of the correlations are not equal to zero at a significance level of < 2.2e-16 (*t*-test), respectively. Then, we compared the numbers of DEGs called by *a*GRP and the previous methods at an ad hoc BH-adjusted *p*-value cutoff of 0.01, as shown in Fig. 2b. From this figure, we can see that *a*GRP still called more DEGs than those by previous methods, GRP (0.9), Rankprod, DEGSeq, DESeq2 and edgeR, on the RNA-Seq data, consistent with the results on the simulation gene expression and the three lung cancer microarray data, confirming the especial power of *a*GRP in identifying subtle but consistent expression changes. Among the 7234 DEGs identified by *a*GRP, there are totally 3548 (Additional file 5: Table S4) and 3686 (Additional file 6: Table S5) with positive *a*GRP statistics and negative *a*GRP statistics, respectively. Literature survey shows that many of these genes are associated with HCC or other types of cancer. Among the 3548 positive *a*GRP DEGs, for example, MMS19 (*a*GRP = 0.69) is a DNA repair gene playing important role in Nucleotide Excision Repair (NER) pathway, whose single nucleotide polymorphism, rs3740526 has been reported to significantly distinguish adenocarcinoma with squamous cell carcinoma and whose expression levels are clinically related with ACT benefit of resected non-small cell lung cancer patients [43, 44]. TRIB1 (*a*GRP = 0.66) has been previously evidenced to be associated with tumorigeneses of various types of cancer, e.g., leukemia and colorectal cancer [45, 46]. Especially, Gendelman et al... [47] computationally inferred that TRIB1 is potentially a regulator of cell-cycle progression and survival in cancer cells and experimentally observed that the expression of TRIB1 is predictive of clinical outcome of breast cancer. DDX59 (*a*GRP = 0.645) has been extensively observed to be highly expressed in lung adenocarcinoma and promote DNA replication in lung cancer development [48, 49]. In addition, among the 3686 negative *a*GRP DEGs, hormone receptor PGRMC2 (*a*GRP = − 0.635) was previously reported to be a tumor suppressor and an inhibitor of migration of cancer cell [50]. Recently, Causey et al [51] also observed that the expression level of PGRMC2 is informative in clinically staging breast cancer and is potentially useful to distinguish low stage tumors from higher stages.

## Discussion

Currently, the expression heterogeneity remains challenging in transcriptomics data analysis. Ignoring the heterogeneity often leads to inconsistent and non-reproducible identification of cancer biomarkers across studies. To our knowledge, there do not exist computational models that are dedicated to address the problem of expression heterogeneity. Compared with previous methods, aGRP operates in a regulation space but not in the expression space. This makes it possible to interrogate and adaptively capture the inter- or intra-class heterogeneity of expression for biologically meaningful identification of cancer biomarkers, as demonstrated in experiments on two types of simulation data (Fig. 1 and Table 1). The advantage endows aGRP with the power of detecting more subtle but consistent DEGs across the three real-world lung cancer data sets (Figs. 2 and 3). We hope that this work can encourage researchers to take advantage of prior knowledge on gene regulation in transcriptional data analysis.

## Conclusions

In this paper, we have presented a novel computational method, *aGRP*, for cancer biomarker identification. It aims to deal with the problem of expression heterogeneity that complicates the identification of cancer biomarkers. Specifically, two regulation events were defined between tumor and normal tissues, whose occurring probabilities were estimated in an unbiased way, and were used to transform the expression profiles of samples to a regulation profile. With the regulation profiles, a new GRP-based statistic were finally formulated for characterizing different expression of genes along with an asymptotic estimator of significance. aGRP automatically interrogates and adaptively captures the heterogeneity of gene expression so that subtle but consistent expression changes can be detected in a flexible and robust way. aGRP is also simple and easy to use in practice. Comparison experiments with current state-of-the-art methods on two simulation data sets and three real-world lung cancer expression data sets and one RNA-seq HCC data set demonstrated the effectiveness and efficiency of *aGRP* in identifying cancer biomarkers from transcriptomics data. Future work will be extending the proposed method to assessment of the heterogeneity of gene sets and meta-analysis of multiple transcriptomics data sets for cancer biomarker identification.

## Additional files

Additional file 1: A proof of the significance estimator of aGRP and three supplemental figures (**Figures S1-S2**). (DOCX 880 kb)

Additional file 2: **Table S1.** List of 1104 DEGs identified on the three lung cancer data sets with negative *a*GRP statistics. (XLS 327 kb)

Additional file 3: **Table S2.** List of 869 DEGs identified on the three lung cancer data sets with positive *a*GRP statistics. (XLS 263 kb)

Additional file 4: **Table S3.** List of 38 KEGG pathways that are reported by DAVID to significantly enriched in the DEG list. (XLS 45 kb)

Additional file 5: **Table S4.** List of 3548 DEGs identified on the HCC RNA-Seq data set with positive *a*GRP statistics. (CSV 194 kb)

Additional file 6: **Table S5.** List of 3686 DEGs identified on the HCC RNA-Seq data set with negative *a*GRP statistics. (CSV 205 kb)

## Abbreviations

aGRP: Adaptive GRP model
DEGs: Differentially expressed genes
FC: Fold-change
GRPs: Gene regulation probabilities
HCC: Hepatocellular carcinoma
LUAD: Lung adenocarcinoma
NSCLC: Non-small cell lung cancer (NSCLC)
NTL: Normal tissue of lung
ROC: The receiver operating characteristic curve

## References

[1] Baker S. G. (2014). A Cancer Theory Kerfuffle Can Lead to New Lines of Research. JNCI Journal of the National Cancer Institute.

[2] Ghazani AA, Oliver NM, St. Pierre JP, Garofalo A, Rainville IR, Hiller E, Treacy DJ, Rojas-Rudilla V, Wood S, Bair E, et al. Assigning clinical meaning to somatic and germ-line whole-exome sequencing data in a prospective cancer precision medicine study. Genet Med. 2017. 10.1038/gim.2016.191 28125075

[3] Goodenberger ML, Jenkins RB (2012). Genetics of adult glioma. Cancer Genetics.

[4] Switnicki M, Juul M, Madsen T, Sorensen KD, Pedersen JS (2016). PINCAGE: probabilistic integration of cancer genomics data for perturbed gene identification and sample classification. Bioinformatics.

[5] Ganjali M, Baghfalaki T, Berridge D (2015). Robust modeling of differential gene expression data using Normal/independent distributions: a Bayesian approach. PLoS One.

[6] Strbenac D, Mann GJ, Yang JYH, Ormerod JT (2016). Differential distribution improves gene selection stability and has competitive classification performance for patient survival. Nucleic Acids Res.

[7] Bae K, Mallick BK (2004). Gene selection using a two-level hierarchical Bayesian model. Binformatics.

[8] Smyth GK. Limma: linear models for microarray data. In: Gentleman R, Carey VJ, Huber W, Irizarry RA, Dudoit S. (eds). Bioinformatics and Computational Biology Solutions using R and Bioconductor. Statistics for Biology and Health. New York: Springer; 2005.

[9] Breitling R, Herzyk P (2005). Rank-based methods as a non-parametric alternative of the T-statistic for the analysis of biological microarray data. J Bioinforma Comput Biol.

[10] Trapnell C, Roberts A, Goff L, Pertea G, Kim D, Kelley DR, Pimentel H, Salzberg SL, Rinn JL, Pachter L (2012). Corrigendum: differential gene and transcript expression analysis of RNA-seq experiments with TopHat and cufflinks. Nat Protocols.

[11] Anders S, Huber W (2010). Differential expression analysis for sequence count data. Genome Biol.

[12] Wang L, Feng Z, Wang X, Wang X, Zhang X (2010). DEGseq: an R package for identifying differentially expressed genes from RNA-seq data. Bioinformatics.

[13] Robinson MD, McCarthy DJ, Smyth GK (2009). edgeR: a Bioconductor package for differential expression analysis of digital gene expression data. Bioinformatics.

[14] Tusher VG, Tibshirani R, Chu G (2001). Significance analysis of microarrays applied to the ionizing radiation response. PNAS.

[15] Hong F, Breitling R (2008). A comparison of meta-analysis methods for detecting differentially expressed genes in microarray experiments. Bioinformatics.

[16] Nabavi S, Schmolze D, Maitituoheti M, Malladi S, Beck AH (2015). EMDomics: a robust and powerful method for the identification of genes differentially expressed between heterogeneous classes. Bioinformatics.

[17] Jiang Y, Qiu Y, Minn A, Zhang N (2016). Assessing intratumor heterogeneity and tracking longitudinal and spatial clonal evolutionary history by next-generation sequencing. Pro Natl Acad Sci USA.

[18] Burrell RA, McGranahan N, Bartek J, Swanton C (2013). The causes and consequences of genetic heterogeneity in cancer evolution. Nature.

[19] Wang H-Q, Huang D-S (2006). Regulation probability method for gene selection. Pattern Recognition Letter.

[20] Gendoo DMA, Ratanasirigulchai N, Schrader MS, Paro L, Parker JS, Prat A, Haibe-Kains B (2015). Genefu: an R/Bioconductor package for computation of gene expression-based signatures in breast cancer. Bioinformatics.

[21] Wang H-Q, Tuominen LK, Tsai C-J (2011). SLIM: a sliding linear model for estimating the proportion of true null hypotheses in datasets with dependence structures. Bioinformatics.

[22] Hong F, Breitling R, McEntee CW, Wittner BS, Nemhauser JL, Chory J (2006). RankProd: a bioconductor package for detecting differentially expressed genes in meta-analysis. Bioinformatics.

[23] Takakura S, Kohno T, Manda R, Okamoto A, Tanaka T, Yokota J (2001). Genetic alterations and expression of the protein phosphatase 1 genes in human cancers. Int J Oncol.

[24] Luo W, Xu C, Ayello J, Dela Cruz F, Rosenblum JM, Lessnick SL, Cairo MS (2017). Protein phosphatase 1 regulatory subunit 1A in Ewing sarcoma tumorigenesis and metastasis. Oncogene.

[25] Hu S, Danilov AV, Godek K, Orr B, Tafe LJ, Rodriguezcanales J, Behrens C, Mino B, Moran CA, Memoli VA (2015). CDK2 inhibition causes anaphase catastrophe in lung Cancer through the Centrosomal protein CP110. Cancer Res.

[26] Zhang Y, Wang Y, Wei Y, Li M, Yu S, Ye M, Zhang H, Chen S, Liu W, Zhang J (2015). MiR-129-3p promotes docetaxel resistance of breast cancer cells via CP110 inhibition. Sci Rep.

[27] Bijnsdorp IV, Jasmina H, Tonny L, Bart W, Oscar K, Jurjen B, Frederik V, Nilsson RJA, Lawrence R, Van BVW (2016). miR-129-3p controls centrosome number in metastatic prostate cancer cells by repressing CP110. Oncotarget.

[28] Fujitomo T, Daigo Y, Matsuda K, Ueda K, Nakamura Y (2014). Identification of a nuclear protein, LRRC42, involved in lung carcinogenesis. Int J Oncol.

[29] Smith LT, Lin M, Brena RM, Lang JC, Schuller DE, Otterson GA, Morrison CD, Smiraglia DJ, Plass C (2006). Epigenetic regulation of the tumor suppressor gene TCF21 on 6q23-q24 in lung and head and neck cancer. Pro Natl Acad Sci USA.

[30] Wang H-Q, Zheng C-H, Zhao X-M (2015). jNMFMA: a joint non-negative matrix factorization meta-analysis of transcriptomics data. Bioinformatics.

[31] Richards KL, Zhang B, Sun M, Dong W, Churchill J, Bachinski LL, Wilson CD, Baggerly KA, Yin G, Hayes DN (2010). Methylation of the candidate biomarker TCF21 is very frequent across a spectrum of early-stage nonsmall cell lung cancers. Cancer.

[32] Shivapurkar N, Stastny V, Xie Y, Prinsen C, Frenkel E, Czerniak B, Thunnissen FB, Minna JD, Gazdar AF (2008). Differential methylation of a short CpG-rich sequence within exon 1 of TCF21 gene: a promising Cancer biomarker assay. Cancer Epidemiol Biomarkers Prev.

[33] Shen L, Yang M, Lin Q, Zhang Z, Zhu B, Miao C (2016). COL11A1 is overexpressed in recurrent non-small cell lung cancer and promotes cell proliferation, migration, invasion and drug resistance. Oncol Rep.

[34] Wu YH, Chang TH, Huang YF, Huang HD, Chou CY (2013). COL11A1 promotes tumor progression and predicts poor clinical outcome in ovarian cancer. Oncogene.

[35] Zhang Z, Wang Q, Chen F, Liu J (2014). Elevated expression of HMGA1 correlates with the malignant status and prognosis of non-small cell lung cancer. Tumor Biol.

[36] Wang L, Zhao L, Qiao Y (2016). Identification of potential therapeutic targets for lung cancer by bioinformatics analysis. Mol Med Rep.

[37] Tang J-M, He Q-Y, Guo R-X, Chang X-J (2006). Phosphorylated Akt overexpression and loss of PTEN expression in non-small cell lung cancer confers poor prognosis. Lung Cancer.

[38] Zhang Y, Bao C, Mu Q, Chen J, Wang J, Mi Y, Sayari AJ, Chen Y, Guo M (2016). Reversal of cisplatin resistance by inhibiting PI3K/Akt signal pathway in human lung cancer cells. Neoplasma.

[39] Yang M, Wang H, Zhou M, Liu W, Kuang P, Liang H, Yuan Q (2016). The natural compound sulforaphene, as a novel anticancer reagent, targeting PI3K-AKT signaling pathway in lung cancer. Oncotarget.

[40] Wang P, Liu N, Pang Q, Qu C, Wang B, Guo H (2012). PI3K/AKT signaling pathway in the regulation of non-small cell lung Cancer Radiosensitivity after Hypofractionated radiation therapy. Int J Radiat Oncol Biol Phys.

[41] Yang Y, Chen L, Gu J, Zhang H, Yuan J, Lian Q, Lv G, Wang S, Wu Y, Yang YT (2017). Recurrently deregulated lncRNAs in hepatocellular carcinoma. Nat Commun.

[42] Love MI, Huber W, Anders S (2014). Moderated estimation of fold change and dispersion for RNA-seq data with DESeq2. Genome Biol.

[43] Adam J, Sourisseau T, Olaussen KA, Robin A, Zhu CQ, Templier A, Civet A, Girard P, Lazar V, Validire P (2016). MMS19 as a potential predictive marker of adjuvant chemotherapy benefit in resected non-small cell lung cancer. Cancer Biomarkers.

[44] Bing LI, Xue-Fei LI (2011). Relationship between DNA repair gene MMS19 single nucleotide polymorphisms and histology of non-small cell lung cancer. Tumor.

[45] Wang Y, Nan W, Bo P, Tong D, Sun D, Sun H, Zhang C, Sun W, Meng X, Jing B (2017). TRIB1 promotes colorectal cancer cell migration and invasion through activation MMP-2 via FAK/Src and ERK pathways. Oncotarget.

[46] Takuro N (2015). The role of Trib1 in myeloid leukaemogenesis and differentiation. Biochem Soc Trans.

[47] Gendelman R, Xing H, Mirzoeva OK, Sarde P, Curtis C, Feiler HS, Mcdonagh P, Gray JW, Khalil I, Korn WM (2017). Bayesian network inference modeling identifies TRIB1 as a novel regulator of cell-cycle progression and survival in Cancer cells. Cancer Res.

[48] You J, Wang X, Wang J, Yuan B, Zhang Y (2017). DDX59 promotes DNA replication in lung adenocarcinoma. Cell Death Discovery.

[49] Yang L, Zhang H, Chen D, Ding P, Yuan Y, Zhang Y (2017). EGFR and Ras regulate DDX59 during lung cancer development. Gene..

[50] Wendler A, Wehling M (2013). PGRMC2, a yet uncharacterized protein with potential as tumor suppressor, migration inhibitor, and regulator of cytochrome P450 enzyme activity. Steroids.

[51] Causey MW, Huston LJ, Harold DM, Charaba CJ, Ippolito DL, Hoffer ZS, Brown TA, Stallings JD (2011). Transcriptional analysis of novel hormone receptors PGRMC1 and PGRMC2 as potential biomarkers of breast adenocarcinoma staging. J Surg Res.

