# Supplementary material for: Adaptively capturing the heterogeneity of expression for cancer biomarker identification
Source: BMC Bioinformatics. 2018 Nov 3;19:401. doi: 10.1186/s12859-018-2437-2 (PMC6215657; doi:10.1186/s12859-018-2437-2)
Supplement: Supplementary file 1 — A proof of the significance estimator of aGRP and three supplemental figures (Figures S1-S2). (DOCX 880 kb) [file 12859_2018_2437_MOESM1_ESM.docx]

## A proof of the null distribution of the *aGRP* statistic under normal distribution data

Assuming that two groups of samples comes from two normal distributions, $Y_{1}\sim\left(\mu_{1},\sigma_{1}^{2}\right)$ and $Y_{2}\sim\left(\mu_{2},\sigma_{2}^{2}\right)$, and have $\left(Y_{1}-Y_{2}\right)\sim N\left(\mu_{1}-\mu_{2},\sigma_{1}^{2}+\sigma_{2}^{2}\right)$, we have

where $\varphi\left(x\right)=\int_{-\infty}^{x} \frac{1}{\sqrt{2\pi}}e^{-\frac{t^{2}}{2}}dt$.

Let $q=\varphi\left(\frac{\mu_{1}-\mu_{2}}{\sqrt{\sigma_{1}^{2}+\sigma_{2}^{2}}}\right)$，then $b_{i}^{u1}=\sum_{k=1}^{m} I(a_{1i}\geq a_{2k})\sim B\left(m,q\right)$. According to the Laplace theorem, $b_{i}^{u1}$ asymptotically follows a normal distribution, $b_{i}^{u1}\sim N\left(mp,mp\left(1-q\right)\right)$. Then, the quantities in Equations (9)-(10) in the main text $\sum_{i=1}^{n} l_{i}=\sum_{i=1}^{n} (\sum_{k=1}^{m} I(a_{1i}\geq a_{2k}))/m=\sum_{i=1}^{n} b_{i}^{u1}/m$ follow the following normal distributions:$\sum_{i=1}^{n} l_{i}\sim N\left(np,np\left(1-q\right)/m\right)$, and $\sum_{i=1}^{n} (1-l_{i})\sim N\left(n\left(1-p\right),np\left(1-q\right)/m\right)$. Likewise, we have $\sum_{i=1}^{n} k_{i}\sim N\left(mp,mp\left(1-q\right)/n\right)$ and $\sum_{i=1}^{n} (1-l_{i})\sim N\left(n\left(1-p\right),np\left(1-q\right)/m\right)$. Therefore, the two regulation probabilities in Equation (9) in the main text, $P\left(U\right)=\frac{\sum_{i=1}^{n} l_{i}+\sum_{j=1}^{m} k_{j}}{n+m}$and $P\left(D\right)=\frac{\sum_{i=1}^{n} (1-l_{i})+\sum_{j=1}^{m} (1-k_{j})}{n+m}$, follow the following normal distributions,

$P\left(U\right)\sim N\left(q,\frac{\left(n^{2}+m^{2}\right)q\left(1-q\right)}{nm\left(n+m\right)^{2}}\right)$ and $P\left(D\right)\sim N\left(1-q,\frac{\left(n^{2}+m^{2}\right)q\left(1-q\right)}{nm\left(n+m\right)^{2}}\right)$, respectively. And the aGRP statistic, $\frac{2}{n+m}\left(\sum_{i=1}^{n} l_{i}+\sum_{j=1}^{m} k_{j}\right)-1$, in Equation (10) in the main text follows a normal distribution:

$$N\left(\mu,\sigma^{2}\right)$$

where $\mu=2p-1$ and $\sigma^{2}=\frac{2\left(n^{2}+m^{2}\right)q\left(1-q\right)}{nm\left(n+m\right)^{2}}$.

Under the null hypothesis *H*_0_:$\mu_{1}=\mu_{2}$, we have$q=\varphi\left(\frac{\mu_{1}-\mu_{2}}{\sqrt{\sigma_{1}^{2}+\sigma_{2}^{2}}}\right)$=0.5,$\mu=0$, and $\sigma^{2}=\frac{\left(n^{2}+m^{2}\right)}{2nm\left(n+m\right)^{2}}$. Therefore, the aGRP statistic has the following null distribution

$$N\left(0,\frac{\left(n^{2}+m^{2}\right)}{2nm\left(n+m\right)^{2}}\right)$$

## Fig.S1 Scatter plot of *P*(*U*) and *P*(*D*) by *aGRP* or GRP with *τ*=0.5, 0.7, 0.9 on Simple simulation data


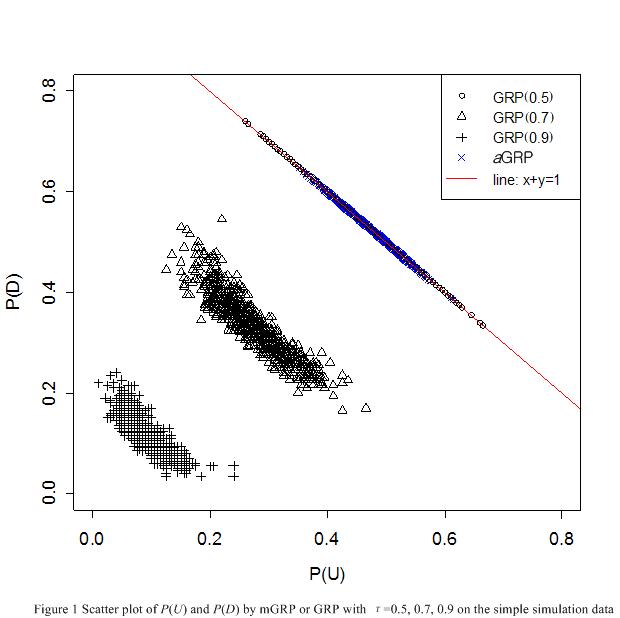


## Fig.S2 Scatter plots of *p*-value estimates for *aGRP* by the asymptotical significance estimator and conventional permutation tests with permutation time settings of *B*=10 (A), 50(B), 100(C), 1000(D) on Simple simulation data.
